# A Single-Domain Antibody Targeting Complement Component C5 Acts as a Selective Inhibitor of the Terminal Pathway of the Complement System and Thus Functionally Mimicks the C-Terminal Domain of the *Staphylococcus aureus* SSL7 Protein

**DOI:** 10.3389/fimmu.2018.02822

**Published:** 2018-11-29

**Authors:** Laure Yatime, Nicolas S. Merle, Annette G. Hansen, Niels Anton Friis, Jakob A. Østergaard, Mette Bjerre, Lubka T. Roumenina, Steffen Thiel, Peter Kristensen, Gregers R. Andersen

**Affiliations:** ^1^Department of Molecular Biology and Genetics, Aarhus University, Aarhus, Denmark; ^2^Centre de Recherche des Cordeliers, INSERM, UMR_S 1138, Paris, France; ^3^Department of Biomedicine, Aarhus University, Aarhus, Denmark; ^4^The Medical Research Laboratory, Department of Clinical Medicine, Endocrinology and Internal Medicine, Aarhus University Hospital, Aarhus University, Aarhus, Denmark; ^5^Department of Chemistry and Bioscience, Aalborg University, Aalborg, Denmark

**Keywords:** complement, terminal pathway inhibition, single-domain antibody, pathogen mimicry, hemolysis, bacteriolysis, paroxysmal nocturnal hemoglobinuria

## Abstract

The complement system is an efficient anti-microbial effector mechanism. On the other hand abnormal complement activation is involved in the pathogenesis of multiple inflammatory and hemolytic diseases. As general inhibition of the complement system may jeopardize patient health due to increased susceptibility to infections, the development of pathway-specific complement therapeutics has been a long-lasting goal over the last decades. In particular, pathogen mimicry has been considered as a promising approach for the design of selective anti-complement drugs. The C-terminal domain of staphylococcal superantigen-like protein 7 (SSL7), a protein secreted by *Staphylococcus aureus*, was recently found to be a specific inhibitor of the terminal pathway of the complement system, providing selective inhibition of cell lysis mediated by the membrane attack complex (MAC). We describe here the selection by phage display of a humanized single-domain antibody (sdAb) mimicking the C-terminal domain of SSL7. The antibody, called sdAb_E4, binds complement C5 with an affinity in the low micromolar range. Furthermore, sdAb_E4 induces selective inhibition of MAC-mediated lysis, allowing inhibition of red blood cell hemolysis and inhibition of complement deposition on apopto-necrotic cells, while maintaining efficient bactericidal activity of the complement terminal pathway. Finally, we present preliminary results indicating that sdAb_E4 may also be efficient in inhibiting hemolysis of erythrocytes from patients with paroxysmal nocturnal hemoglobinuria. Our data provide a proof of concept for the design of a selective MAC inhibitor capable of retaining complement bacteriolytic activity and this study opens up promising perspectives for the development of an sdAb_E4-derived therapeutics with application in the treatment of complement-mediated hemolytic disorders.

## Introduction

Upon detection of pathogenic or damaged cells in our body, the complement cascade of the innate immunity is activated. It results in a complex chain of proteolytic reactions that ultimately leads to cleavage of the complement factor C5 and initiation of the terminal pathway (TP) of the complement system (1, 2). This cleavage generates the small C5a anaphylatoxin, which triggers the inflammatory response, and the large C5b fragment, which then recruits complement proteins C6-C9 to form the membrane attack complex (MAC) responsible for the lysis of the targeted cells (3–5). Whereas this process is crucial for host defense upon pathogenic invasion, it has to be tightly regulated to prevent aberrant complement activation in non-infected areas and the ensuing risk of damaging healthy tissues. For this purpose, host cells express membrane-bound and soluble regulators that efficiently block the complement cascade (6, 7). Dysregulation of the complement system, often linked to genetic deficiencies in these regulatory factors, can lead to various pathological conditions, including inflammatory and hemolytic disorders that result from an excessive C5a production and/or the targeting of host red blood cells by the MAC (1, 4, 8, 9).

A crucial role of complement component C5 has been demonstrated for several pathologies, e.g., the two life-threatening hemolytic diseases paroxysmal nocturnal hemoglobinuria (PNH) (10) and atypical hemolytic uremic syndrome (aHUS) (11). PNH arises from mutations in the PIG-A gene in bone marrow stem cells. The resulting matured cells, including erythrocytes, are deficient in GPI-anchored proteins and, as a result, lack the MAC inhibitor CD59, which leads to their destruction by the complement lytic complex (12, 13). aHUS is characterized by renal-specific thrombotic microangiopathy which causes massive organ failure and possibly death without specific treatment (14). C5 activation also plays a key role in the pathogenesis of aHUS although the respective contribution of both C5a-mediated inflammation and uncontrolled MAC activation on endothelial cell surface is still unclear (11). Due to its critical involvement in PNH and aHUS, C5 has been identified as an important drug target for the treatment of both diseases (15, 16).

Eculizumab, one of the rare complement inhibitors available on the market, is a monoclonal anti-C5 antibody approved for the treatment of PNH and aHUS (15). As a general C5 inhibitor, it efficiently reduces RBC lysis and endothelial damage, thereby improving considerably the quality of life of the patients (17, 18). One of the major drawbacks associated with the administration of eculizumab is the increased risk of developing a meningococcal disease since the treated patients lack the lytic MAC for destruction of these bacteria as well as the C5a-dependent activation of phagocytic cells (19, 20). Current recommendations call for vaccination against meningococci prior to eculizumab treatment, but even in these conditions, neisserial infections are still occurring, although to a much lower extent and in a patient-specific manner (19, 21). Thus, more specific C5 inhibitors could be advantageous, in particular inhibitors that would preserve the TP bactericidal activity. At the moment, various drug design strategies are being considered to obtain such molecules (7, 22). In particular, one general approach to obtain complement inhibitors consists in developing molecules that mimic the properties of complement evasion proteins secreted by pathogens in order to prevent complement activation (23). One example is the secreted staphylococcal superantigen-like protein 7 (SSL7) which targets C5 (24). Interestingly, whereas the full-length protein acts as a general C5 inhibitor, thereby mimicking the mode of action of eculizumab, we found that the C-terminal domain of the protein behaves as a selective MAC inhibitor, not only allowing partial C5 cleavage and subsequent C5a release, but also blocking RBC hemolysis while maintaining efficient bacteriolysis (25). Hence, if the SSL7 C-terminal domain (SSL7-CT) was used to block C5 cleavage, the risk of meningococcal disease could be lower than with existing anti-C5 therapeutics. As SSL7-CT is of bacterial origin, it cannot be administered to the human body without risking the activation of the host immune system. To circumvent this problem and still take advantage of the appealing properties of SSL7-CT, we aimed at selecting a single-domain antibody (sdAb) that would have the same behavior toward C5 as SSL7-CT.

In this paper, we describe an innovative structure-based strategy for the selection of anti-C5 sdAbs by phage display using competitive elution, and we present the evaluation of their functional properties with respect to inhibition of C5 cleavage and MAC assembly on erythrocytes, bacteria and endothelial cells. Our data provide a proof of concept for the selection of an anti-C5 sdAb that prevents MAC formation on host cells while maintaining the bactericidal activity of the terminal pathway of complement.

## Materials and methods

### Single-domain antibody selection using phage display and ELISA

A phage display library composed of human single-domain antibodies corresponding to the variable domain of the heavy chain and derived from the HEL4 sdAb (26, 27) by randomization on all three CDR regions (V_H_ sdAbs, Garvan library, https://www.sourcebioscience.com/products/life-sciences-research/clones/artificial-antibody-libraries/human-domain-antibody-library-dab/) was used for the selection (generous gift from Dr. Daniel Christ, Garvan Institute, Australia). The selection was performed according to standard protocols (28) except for the elution step. Briefly, an immunotube was coated overnight at 4°C with 75 μg/ml of plasma-purified human C5 (29) diluted in PBS. Residual binding was blocked by incubation with 2% low fat milk powder in PBS (MPBS). Around 10^12^ sdAb-displaying phages from the Garvan library were then added to the tube and incubated for 2 h at room temperature. After washing off unbound phages with PBST (0.1 % Tween 20 in PBS), elution was performed with a 75 μg/ml solution of purified SSL7-CT. The eluted phages were used to infect *E. coli* TG1 cells in the log phase. Single colonies were picked and the corresponding sdAb-phages were rescued for ELISA using the KM13 phage helper (30).

ELISA was performed in 96-well Nunc MaxiSorp plates coated with 8 μg/ml of human C5. The C5-binding phages were detected with an anti-M13 antibody conjugated to horseradish peroxidase (Pharmacia) followed by development with TMB substrate/sulfuric acid and reading of the absorbance at 450 nm on a Bio-Rad Model 550 microplate reader. 50 ml phage cultures were prepared for the ELISA-positive clones. Following precipitation of the phages with PEG (20 % polyethylene glycol 6000 and 2.5 M NaCl), the phage particles were resuspended in PBS and phage dilution series ranging from 10^12^ to 0.5 × 10^9^ phages/ml were tested again in the C5-ELISA. The DNA sequences for the three best clones were obtained from MWG Eurofins (Germany) (DNA sequences available upon request).

### Purification of the V_H_ sdAbs, SSL7-CT and complement C5

The sequences coding for the V_H_ sdAbs selected by phage display were cloned into the *Nco* I and *Xho* I restriction sites of vector pETM11 (EMBL vector collection). A stop codon was added just before the *Xho* I site in the reverse primer used for PCR amplification. The resulting constructs were transformed in *E. coli* BL21 (DE3) cells (New England Biolabs). Cells were grown for 4 h at 37°C in 2xYT medium supplemented with kanamycin and protein expression was induced overnight at 18°C by the addition of 1 mM IPTG. Bacterial pellets were harvested and sonicated in Buffer A (50 mM HEPES pH 7.5, 300 mM NaCl, 30 mM imidazole, 1 mM PMSF). After clarification by centrifugation, the supernatant was applied onto a 5 mL HisTrap FF column (GE Healthcare) equilibrated in Buffer A. After high salt wash (50 mM HEPES pH 7.5, 1 M NaCl, 30 mM imidazole, 1 mM PMSF) to remove unspecifically bound proteins, the His_6_-tagged proteins were eluted with Buffer B (50 mM HEPES pH 7.5, 300 mM NaCl, 500 mM imidazole, 1 mM PMSF). The His_6_-tag was then removed by overnight cleavage with in-house prepared recombinant TEV protease at 4°C in buffer C (50 mM HEPES pH 7.5, 300 mM NaCl, 0.5 mM EDTA) followed by a second run on the HisTrap column. The cleaved proteins present in the run-through fraction were finally purified by size exclusion chromatography (SEC) on a 24 ml Superdex 75 10/300 GL column (GE Healthcare) equilibrated in Buffer D (20 mM HEPES pH 7.5, 150 mM NaCl). Fractions containing the target protein were analyzed by SDS-PAGE, pooled, flash-frozen in liquid nitrogen and stored at −80°C until use.

SSL7 (full-length) and SSL7-CT from strain ATCC 12598 were purified as previously described (25). Briefly, both proteins were expressed in *E. coli* BL21 (DE3) cells. SSL7 was purified using the same protocol as for the V_H_ sdAbs whereas SSL7-CT was expressed with a non-cleavable C-terminal His_6_-tag and was therefore only run once through the HisTrap FF column before SEC. Human C5 was purified from human plasma as previously described (29).

### Detection of soluble C5b-9 (sC5b-9) and C5a in human serum

Formation of plasma sC5b-9 was measured using an in-house immunoassay previously described (31). Briefly, for each measurement point, normal human serum (NHS, with reference concentration for C5 of 75 μg/ml) was activated by incubation for 4 h at 37°C in the absence or in the presence of a 1:1 (SSL7-CT and sdAb_E4, corresponding to a 14-fold molar excess) or 3:1 (SSL7, 21-fold molar excess) mass ratio of inhibitor. A few samples of NHS without inhibitor were kept at 4°C (“no activation” samples). All samples were then diluted in PBST containing 10 mM EDTA and transferred into a 96-wells microplate coated with a monoclonal antibody against human sC5b-9 (Quidel). Following overnight incubation at 4°C, bound sC5b-9 was detected with 0.05 μg/ml of biotinylated anti-human C6 antibody (Quidel) for 2 h at room temperature and was subsequently incubated with 10 ng of Eu^3+^-labeled streptavidin (Perkin Elmer) in 100 μl of PBST containing 25 μM EDTA for 1 h at room temperature. After wash, bound Europium was detected by the addition of 200 μl of enhancement solution (Perkin Elmer) and reading of the time-resolved fluorescence on a DELFIA fluorometer (Victor3, Perkin Elmer) using excitation at 340 nm and recording of the emission at 615 nm. A standard was made from NHS activated by incubation with human Ig-Sepharose for 1 h at 37°C and the concentration of sC5b-9 was quantified by comparison with recombinant sC5b-9 (Quidel). Wells receiving only buffer were used to derive a background value that was subtracted from all sample values.

The concentration of C5a in human serum incubated at 37°C in the presence or in the absence of C5 inhibitors was measured by the use of a sandwich-type ELISA kit from Hycult Biotech (HK349-02). Residual C5a release was estimated from samples before activation at 37°C and this background value was subtracted to all sample values obtained after activation at 37°C.

### Size exclusion chromatography (SEC) binding assay

SEC experiments were performed at 4°C on a 24 ml Superose 6 Increase 10/300 GL column (GE Healthcare) equilibrated in 20 mM HEPES pH 7.5, 150 mM NaCl. 150 μg of purified C5 were incubated with or without a 10-fold molar excess of purified sdAb_E4 for 1.5 h at 37°C before injection onto the SEC column. A third run was performed with sdAb_E4 alone present in the same amount as when incubated with C5. For all runs, fractions corresponding to the position of the C5 peak were analyzed by SDS-PAGE.

### Affinity measurements using microscale thermophoresis (MST) and biolayer interferometry (BLI)

Human C5 was fluorescently labeled using the Monolith NT™Protein Labeling Kit BLUE-NHS (NanoTemper technologies) according to the manufacturer's protocol. Labeled C5 at a final concentration of 25 nM was incubated with a dilution series of sdAb_E4 (ranging from 250 μM to 7.6 nM) in running buffer (20 mM HEPES pH 7.5, 150 mM NaCl, 0.1% Tween 20) for 2 h at 37°C to allow complex formation. MST measurements were then performed on a Monolith NT.115 apparatus with samples loaded in standard capillaries (NanoTemper technologies). Measurements were recorded at 25°C using the BLUE led at 65% and laser power at 90%. Data were analyzed in GraphPad Prism and the K_D_ value was derived from a sigmoidal dose-response fitting model.

BLI measurements were performed at 30°C on an Octet RED96 machine (ForteBio) using amine reactive second-generation (AR2G) biosensors (ForteBio). sdAb_E4-coated sensors were prepared by dipping the sensors into a solution of sdAb at 50 μg/ml in 10 mM Na acetate pH 5.0 for 600 s. Unbound sdAb was then washed off for 300 s in Buffer A (10 mM HEPES pH 7.5, 150 mM NaCl, 0.1% Tween 20) and the baseline was obtained after 120 s wash in Buffer A. Association with C5 (2-fold dilution series from 40 μM to 312.5 nM in Buffer A) was performed over 900 s and the dissociation phase in the same buffer was monitored for 1,800 s. For each point in the dilution series, binding of C5 in the absence of sdAb_E4 was used as a reference to subtract the non-specific interactions of C5 with the biosensor. Curve fitting was performed with GraphPad Prism using non-linear regression.

### Hemolysis assay on sheep/rabbit erythrocytes

Sheep and rabbit blood in Alsevers solution were purchased from SSI Diagnostica. BI buffer was prepared by adding CaCl_2_ to a final concentration of 2 mM to Veronal Buffer (0.037 mg/ml CaCl_2_, 0.168 mg/ml MgCl_2_, 8.5 mg/ml NaCl, 0.574 mg/ml barbitol, 0.374 mg/ml sodium barbitol) from Lonza. GBI buffer corresponded to BI buffer supplemented with 1 mg/ml gelatin. A final suspension of 6% RBCs in GBI buffer was prepared from both sheep and rabbit blood. To allow CP activation, sheep RBCs were pre-incubated for 30 min at room temperature with a solution of rabbit anti-sheep RBC antibody (Sigma, catalog nr. S1389). Fifteen μl of NHS were diluted in PBS (final volume 80 μl), either alone or in the presence of increasing amounts of C5 inhibitor (C5:inhibitor molar ratios ranging from 1:1 to 1:1,000). BI buffer (120 μl) was added and each sample was incubated for 2 h at 37°C under gentle agitation. The samples were then aliquoted thrice in a 96-well microplate with conical V-bottom (Nunc). Each well received 30 μl of RBC suspension and the plate was incubated for 2 h at 37°C under gentle agitation. The lysis reaction was blocked by adding 100 μl/well of a cold stop solution (0.9% NaCl, 5 mM EDTA). Cells were spun down at 2,000 rpm at 4°C for 10 min and the supernatants were transferred into a new 96-well microplate with flat bottom (Nunc). The absorbance at 405 nm was read on a VICTOR 3 1420 Multilabel Plate Reader (PerkinElmer). The reference of 100% hemolysis was extrapolated from samples in which the RBCs were incubated with NHS in the absence of inhibitor. For each sample, the final read was obtained from averaging over the 3 replicate wells and the overall experiment was done in triplicate.

### Bacteriolysis assay

The bactericidal activity of human serum in the presence of C5 inhibitors was assessed using the BacTiter-Glo™microbial cell viability assay (Promega). Fifteen μl of NHS were incubated in PBS (final volume 80 μl) either alone or in the presence of increasing amounts of C5 inhibitor (C5:inhibitor molar ratios ranging from 1:1 to 1:1,500) for 2 h at 37°C under gentle agitation. In each tube was then added 40 μl of a culture of *E. coli* DH5α cells grown in LB medium at an ^600^OD (optical density at 600 nm) of 0.3. After 2 h of incubation at 37°C, each sample was aliquoted thrice in an opaque 96-well microplate (Nunc). An equivalent volume of BacTiter-Glo^TM^ solution was then added in each well and after 5 min. incubation at room temperature, the luminescence was read on a VICTOR 3 1420 Multilabel Plate Reader (PerkinElmer). The reference of 100% surviving bacteria was extrapolated from samples in which the DH5α cells were incubated with only PBS (no NHS). For each sample, the final read was obtained from averaging over the 3 replicate wells and the overall experiment was done in triplicate.

### HUVEC cell culture and activation assay on HUVEC apopto-necrotic cells

Primary human umbilical vein endothelial cells (HUVEC) were used for experiments until passage 4 as described previously (32–34). HUVEC were cultured in M199 medium (containing 1 mM HEPES, penicillin (100 U/mL) streptomycin (100 U/mL), 0.2 mM L-glutamine) supplemented with 20% FCS, 10% EGM2 (Lonza), 50 μg/mL heparin and 10 μg/mL ECGS. Spontaneously detached cells from a confluent HUVEC monolayer after 2–3 days of culture were recovered and their viability was tested by Annexin-V and DAPI staining. This population consists of a small proportion of apoptotic and a larger proportion of necrotic cells and was used for further experiments. Experiments were performed in M199 medium.

The HUVEC apopto-necrotic cells were collected by centrifugation for 5 min at 800 g and incubated with increasing concentrations of different C5 inhibitors diluted in M199. The cells were then exposed to NHS (AB blood group), as described previously (35, 36). HUVEC were stained with an anti-C5b-9 antibody (kindly provided by Prof. Paul Morgan, Cardiff University) and analyzed by flow cytometry (BD LSR II) using the FlowJo X software.

### Hemolysis assay on PNH human erythrocytes

The efficacy of the C5 inhibitors was tested on PNH RBCs using the Ham's test as adapted from (37). Briefly, fresh RBCs from a PNH patient were incubated at a final hematocrit of 1% for 1 h with 50% NHS supplemented with MgCl_2_ at a final 1.5 mM concentration. AP activation was achieved by lowering the pH, through addition of HCl 1N diluted 1:20 to obtain a final pH ranging between 6.7 and 6.9. The C5 inhibitors were added to the tubes at different concentrations before adding NHS. After 1 h of incubation in a water bath at 37°C under agitation, the percentage of erythrocyte lysis was determined by recording the absorbance at 414 nm.

### Data analysis and statistics

Data analysis and curve fitting were done with GraphPad Prism version 6.03 for Windows, GraphPad Software, La Jolla California USA (www.graphpad.com). Statistical analysis was performed in GraphPad Prism using the Student's *t*-test. *P*-values are as follow: ^*^p < 0.05; ^**^p < 0.01; ^***^p < 0.001; ^****^p < 0.0001.

## Results

### Selection of a SSL7-mimicking sdAb by phage display

**Figure 1:**
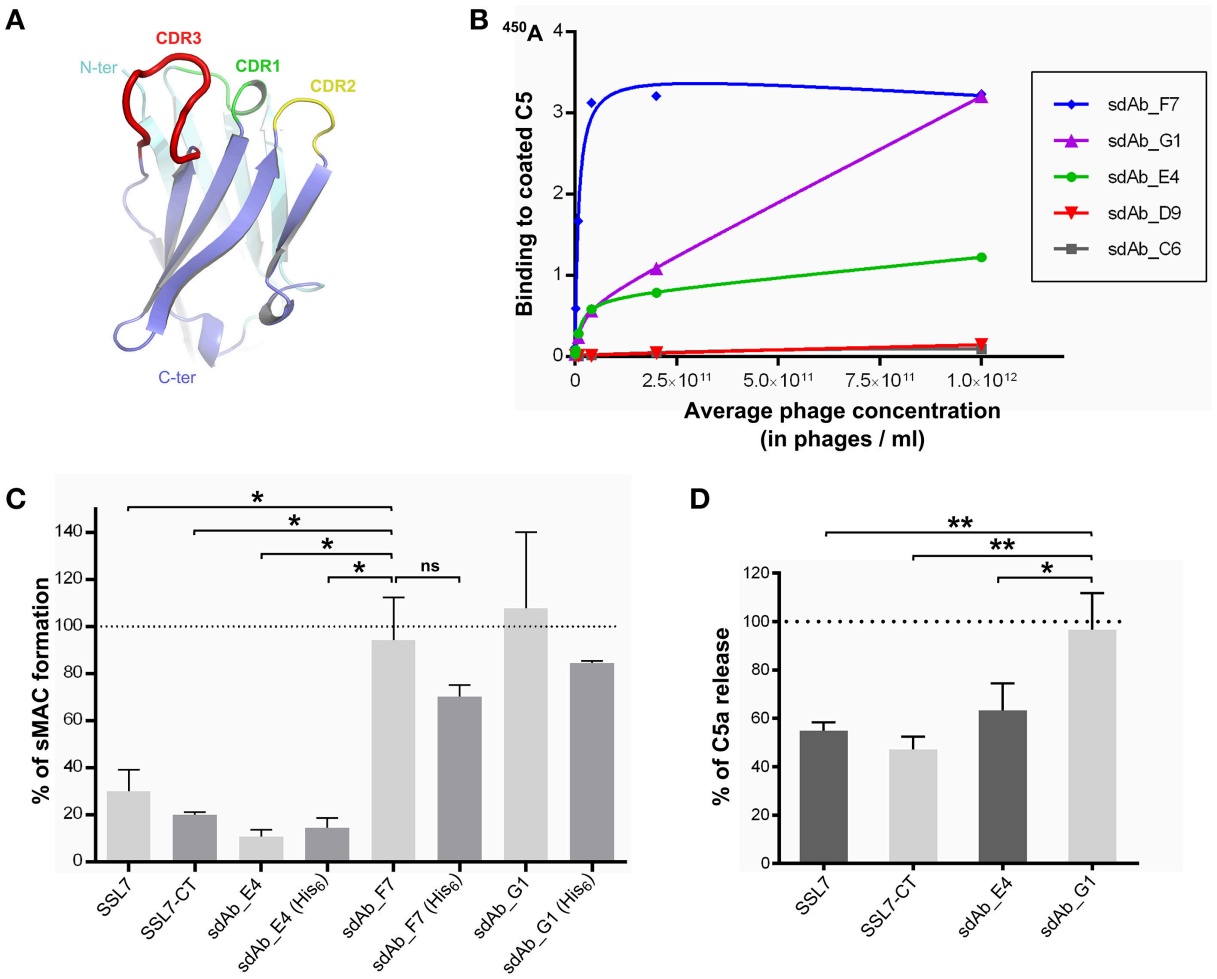
Selection of a MAC inhibitor using phage-displayed single-domain antibodies. **(A)** Structure of the HEL4 domain antibody (26) which constitutes the scaffold for the Garvan library used for phage display selection with CDR regions highlighted in green, yellow and red. **(B)** Results of the ELISA evaluating the binding to C5 of a dilution series of sdAb-displaying phages for the five most promising clones obtained after phage display selection. **(C)** sC5b-9 assay evaluating the formation of soluble C5b-9 complex in human serum upon activation at 37°C, in the absence or in the presence of C5 inhibitors. The presence of a hexahistidine tag on the sdAbs is indicated in parentheses. The 100% reference is taken from NHS activated in the absence of inhibitor. Data points are averaged over two independent measurements. **(D)** C5a assay measuring the amount of C5a released in human serum after activation at 37°C, in the absence or in the presence of C5 inhibitors. The 100% reference is taken from serum activated in the absence of inhibitor. Measurements were done in duplicates in three independent experiments. *P*-values: ^*^p < 0.05; ^**^p < 0.01; ns, difference statistically not significant.

SSL7 is composed of two functionally independent domains (25, 38). Our structure of the C5:SSL7 complex revealed that only the C-terminal β-grasp domain of the protein is required for C5 binding (25), whereas the N-terminal OB domain mediates IgA binding (25, 39). Thus, we hypothesized that in order to obtain an antibody with the same properties as SSL7-CT, i.e., selective inhibition of the MAC, we should search for an antibody recognizing the same epitope as SSL7 on C5. Furthermore, as our structure of the CVF:C5:SSL7 complex revealed that SSL7 interacts with cobra venom factor (CVF), we predicted that with a conventional IgG antibody mimicking SSL7, there would be a significant risk of interfering with C5 binding to the C3b or C4b moiety in the C5 convertases mimicked by CVF in our structure (40). We therefore focused on single-domain antibodies (around 14 kDa) that are comparable in size to SSL7-CT. A library of phages displaying humanized single-domain antibodies corresponding to the variable domain of the heavy chain (V_H_ sdAbs) was therefore used for the selection (Figure 1A). Applying our structure-based strategy, we performed phage display selection on the whole C5 molecule. However, instead of eluting the bound phages with the often used trypsin, we performed a competitive elution with recombinant SSL7-CT. This mode of elution should in principle only displace phages bearing a sdAb with an epitope on C5 overlapping the SSL7 epitope. The eluted sdAb-phages were then tested for direct C5 binding using standard ELISA. Only a small number of positive clones were obtained, which could be due to the fact that only a small fraction of the C5-bound sdAb-phages is displaced by SSL7-CT. Nevertheless, phage dilution series for the most promising clones were evaluated in the C5 ELISA (Figure 1B) and the three most affine clones (sdAb_E4, sdAb_F7 and sdAb_G1) were kept for further analysis.

### sdAb_E4 inhibits soluble C5b-9 formation as well as C5 cleavage

The three selected sdAbs were prepared as recombinant proteins in bacteria. We then tested their ability to block the formation of soluble sC5b-9 in activated human serum. Intact SSL7 and SSL7-CT were used as controls for sC5b-9 inhibition. The sdAbs were produced in two different versions: one with a C-terminal hexahistidine (His_6_) tag and one without any tag. As shown in Figure 1C, both versions of sdAb_E4 inhibited sC5b-9 formation at more than 80% when present at a 14-fold molar excess with respect to C5. The same level of inhibition was achieved by SSL7-CT whereas it was slightly lower with SSL7, even at higher molar excess. The untagged versions of the other sdAbs tested had no effect on sC5b-9 formation (even at a 42-fold molar excess). The tagged versions of these two sdAbs seemed to induce a lower percentage of sC5b-9 assembly perhaps due to unspecific interaction of the His_6_ moiety with C5 or a convertase subunit, but the observed difference with the untagged sdAb_F7 and sdAb_G1 is statistically not significant. In any case, only sdAb_E4 displayed the desired effect.

To evaluate whether sC5b-9 inhibition exerted by sdAb_E4 was due to general inhibition of C5 cleavage or was more specific, we measured the release of C5a in activated human serum in the presence of various C5 inhibitors present at a 15-fold molar excess as compared to C5. As shown in Figure 1D, both SSL7 and SSL7-CT reduced C5a levels in the serum down to almost 50%. When higher concentrations of inhibitor were used (up to 750-fold molar excess), C5 cleavage was impaired by 80-90% by both SSL7 proteins (Supplementary Figure 1). This contrasts with the previous report suggesting that SSL7-CT was able to maintain partial C5 cleavage as compared to intact SSL7 (25). In any case, our selected sdAb acts similarly to SSL7 and SSL7-CT. Indeed, C5 cleavage inhibition reaches 30% when the sdAb is present at a 15-fold molar excess (Figure 1D) and the inhibition increases to 90% at ratios above 1:150 (Supplementary Figure 1). In conclusion, sdAb_E4 is capable of efficiently preventing MAC formation while having an effect on C5 cleavage similar to that of SSL7.

### sdAb_E4 directly binds C5 *in vitro*

**Figure 2:**
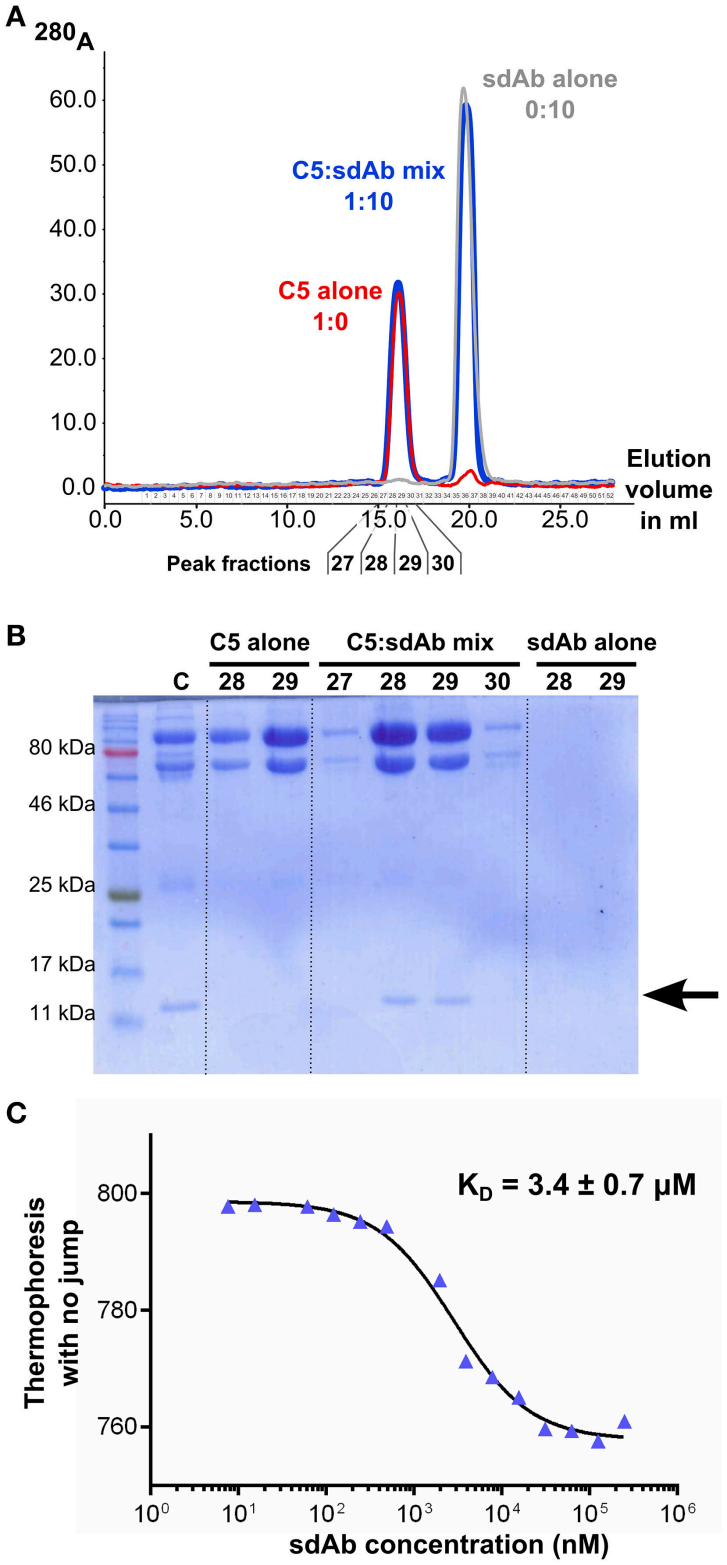
sdAb_E4 binds human C5 *in vitro*. **(A)** Superimposition of the SEC chromatograms obtained with C5 alone (red curve), sdAb_E4 alone (gray curve) or a 1:10 molar mix of C5:sdAb (blue curve) on a Superose 6 column. **(B)** SDS-PAGE analysis of the fractions corresponding to the first peak (higher molecular weight species) in the SEC experiments. Fractions 28 and 29 from all 3 runs were analyzed. The control sample (C) contains purified C5 and sdAb_E4 in a 1:1 molar ratio. **(C)** Microscale thermophoresis data obtained for the binding of sdAb_E4 to labeled C5. sdAb_E4 concentrations are ranging from 250 μM to 7.6 nM (2-fold dilution series). The data points were fitted with a log(inhibitor) vs. response model using GraphPad Prism. A K_D_ value of 3.4 μM was obtained with a quality of the fit *R*^2^ = 0.9895.

To ensure that the properties observed for sdAb_E4 are due to a direct interaction with C5, we then carried out *in vitro* binding assays between sdAb_E4 and plasma-purified C5. First, the formation of a stable complex between the sdAb and its antigen was evaluated using size exclusion chromatography. Figure 2A shows the superimposition of the chromatograms obtained for the two individual components (red and gray curves) and for a mix of the two components in which sdAb_E4 is present at a 10-fold molar excess as compared to C5 (blue curve). The same amount of sdAb_E4 and C5 was used in the runs for the individual proteins as compared to the run for the C5:sdAb mix in order to be able to directly compare the peaks obtained for each run. No clear shift is observed for the C5 peak when the mix is run on the column. This may be explained by considering that the molecular weight of a 1:1 C5:sdAb complex (201.7 kDa) is only slightly higher than that of C5 alone (188.3 kDa). Nevertheless, SDS-PAGE analysis of the fractions eluting between 15 and 17.5 ml (Figure 2B, lanes 5 to 8) reveals that C5 and sdAb_E4 elute together from the column. No band for sdAb_E4 is detected on the gel when the two components are run individually on the column (Figure 2B, lanes 3-4 and 9-10). Thus, a stable complex is formed *in vitro* between human C5 and sdAb_E4 with an apparent 1:1 stoichiometry.

The affinity between sdAb_E4 and human C5 was then measured in solution with microscale thermophoresis, using labeled C5 and increasing concentrations of unlabeled sdAb_E4. MST experiments resulted in K_D_ values ranging between 2 and 6 μM. A typical experiment is displayed in Figure 2C, showing a dose-dependent thermophoretic signal with increasing concentrations of sdAb_E4. Curve fitting with GraphPad Prism gave a K_D_ value of 3.4 ± 0.7 μM (Figure 2C) for this experiment. Affinities in the μM range were also measured with biolayer interferometry (BLI, Supplementary Figure 2). sdAb_E4 was immobilized on the biosensor tip and a dilution series of C5 was used as the analyte in solution. Supplementary Figure 2 shows the BLI curves obtained for a standard experiment. A dose-dependent response of the sdAb-coated biosensor was observed in the presence of increasing amounts of C5, revealing binding of C5 to the biosensor. K_D_ values in the low μM range were obtained for the different series of measurement performed, in good agreement with the values obtained with MST. Thus sdAb_E4 directly binds human C5 *in vitro*, with a medium strength affinity.

### sdAb_E4 prevents erythrocytes hemolysis and MAC deposition on apopto-necrotic cells

**Figure 3:**
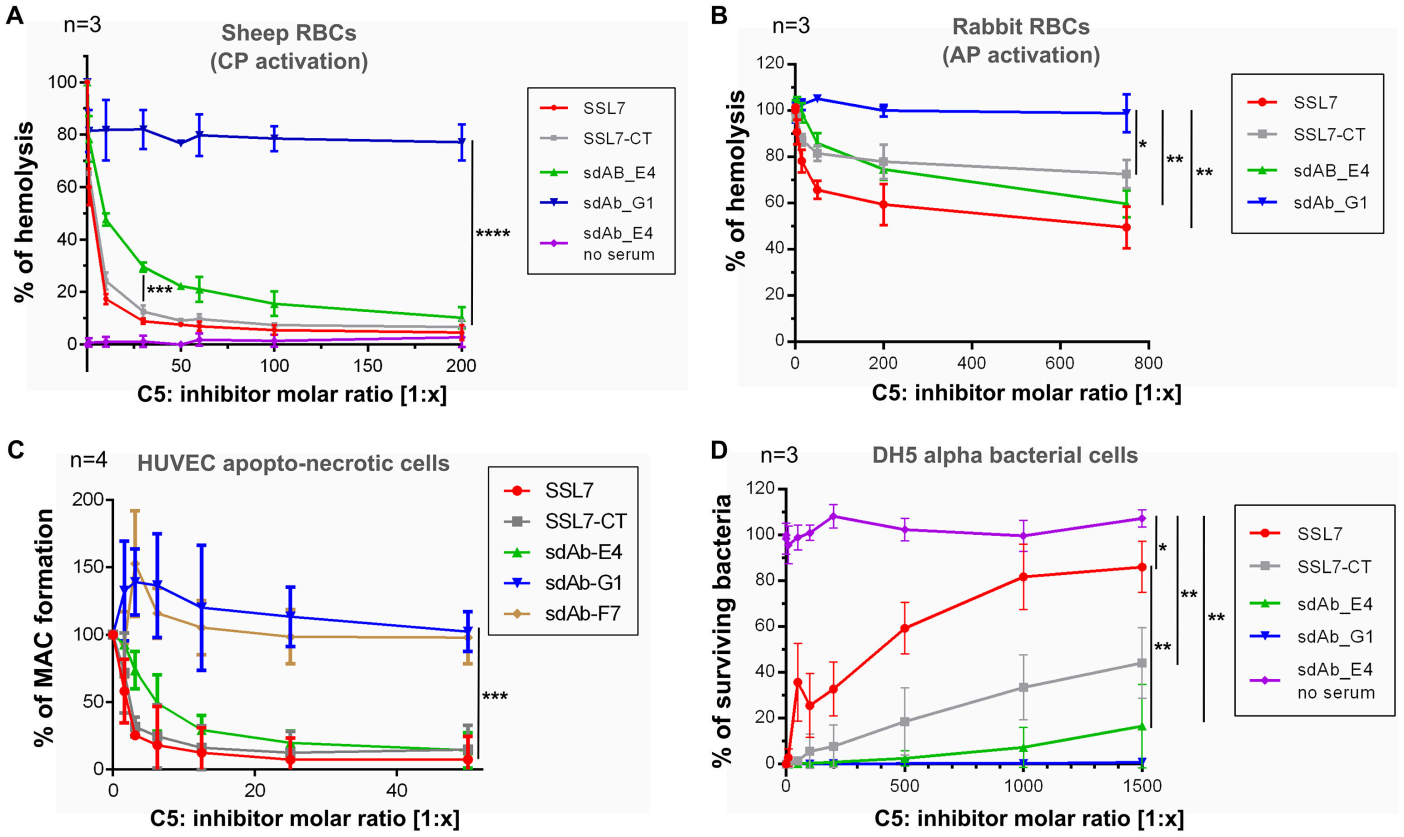
sdAb_E4 prevents RBC hemolysis and MAC deposition on endothelial cells but allows efficient bacteriolysis**. (A)** Hemolysis of sheep RBCs (CP activation) by human serum in the presence of increasing concentrations of C5 inhibitor. The 100% hemolysis reference is taken from sheep RBCs incubated with human serum in the absence of inhibitor. Measurements were done in triplicates in three independent experiments. **(B)** As **(A)** but the hemolysis assay was performed on rabbit RBCs (AP activation). **(C)** MAC formation on apopto-necrotic HUVEC in the presence of increasing concentrations of C5 inhibitor. The 100% reference is taken from endothelial cells incubated with human serum in the absence of inhibitor. Data were averaged over four independent experiments. **(D)** Survival of *E. coli* DH5α cells upon incubation with activated human serum, in the absence or in the presence of increasing concentrations of C5 inhibitor. The 100% survival reference is taken from bacterial cells incubated with PBS, in the absence of NHS. Measurements were done in triplicates in three independent experiments. *P*-values: ^*^p < 0.05; ^**^p < 0.01; ^***^p < 0.001; ^****^p < 0.0001.

To further analyze the mode of action of sdAb_E4, we evaluated its effect on complement-mediated lysis of RBCs using an *in vitro* hemolysis assay. Sheep erythrocytes activated by opsonization with IgG (for testing CP activation) or rabbit erythrocytes (AP activation) were exposed to NHS pre-activated at 37°C in the absence or in the presence of increasing concentrations of C5 inhibitors. In the absence of inhibitors, the sheep and rabbit erythrocytes were massively lysed by NHS (reference value of 100% hemolysis in Figures 3A,B). As expected, SSL7 and SSL7-CT inhibited CP-mediated hemolysis by more than 90%, reaching a plateau at a 1:60 molar ratio as compared to C5 (Figure 3A). sdAb_E4 could also efficiently block RBC lysis although maximum inhibition required higher concentrations of sdAb than for the SSL7 proteins (90% inhibition reached at a 1:500 molar ratio, Figure 3A and Supplementary Figure 3). In contrast, hemolysis was maintained with sdAb_G1, demonstrating that the observed inhibitory effect was specific to sdAb_E4. Inhibition of AP-mediated hemolysis was also achieved by both SSL7, SSL7-CT and sdAb_E4 whereas sdAb_G1 failed to reduce hemolysis of rabbit RBCs (Figure 3B). However, AP-inhibition was only partial as compared to what was observed for the hemolysis assay on sheep RBCs, suggesting the possibility of a differential inhibitory effect depending on which complement pathway is activated.

We then investigated the effect of the anti-C5 sdAb on complement deposition on endothelial cells. Exposure of apopto-necrotic HUVEC to NHS induced complement activation until MAC formation (Figure 3C). Incubation with a 1:12.5 molar ratio of SSL7 completely inhibited MAC formation whereas the same amount of SSL7-CT blocked MAC assembly by more than 90%. In the same manner, sdAb_E4 was able to efficiently inhibit MAC formation and reached the plateau at a 1:25 molar ratio. On the contrary, sdAb_F7 and sdAb_G1 did not decrease MAC formation on apopto-necrotic HUVEC even at a 200-fold molar excess. Taken together these data demonstrate that sdAb_E4 blocks MAC assembly on both endothelial and red blood cells.

### sdAb_E4 maintains efficient bacteriolysis

Since our aim was to obtain a C5 inhibitor with selective properties toward MAC inhibition, we then tested the effect of sdAb_E4 on complement-mediated bacterial killing of *E. coli* DH5α cells. In the absence of inhibitor, human serum completely lysed the bacterial cells (Figure 3D). As shown previously (25), SSL7 prevented bacteriolysis in a dose-dependent manner, reaching a plateau of 80% inhibition at a 1:1,000 molar ratio with C5 (Figure 3D, red curve). In contrast, SSL7-CT only partially blocked the bactericidal activity of NHS with a maximal inhibitory effect of 40% observed at a 1,500-fold molar excess (Figure 3D, gray curve). Interestingly, sdAb_E4 had almost no inhibitory effect on complement-mediated bacteriolysis since bacterial killing remained efficient up to more than 80% at a C5:sdAb molar ratio of 1:1,500 (Figure 3D, green curve). When *E. coli* DH5α cells were incubated with increasing amounts of sdAb_E4 in the absence of serum, all bacteria survived (Figure 3D, purple curve), showing that sdAb_E4 itself has no bacteriolytic activity. The negative control sdAb_G1 had no effect on bacteriolysis (Figure 3D, blue curve). In conclusion, sdAb_E4 allows complement TP lytic activity on bacterial cells, even at a high molar excess, therefore proving to be a MAC selective inhibitor even more efficient than SSL7-CT.

### sdAb_E4 may inhibit hemolysis of human PNH erythrocytes

**Figure 4:**
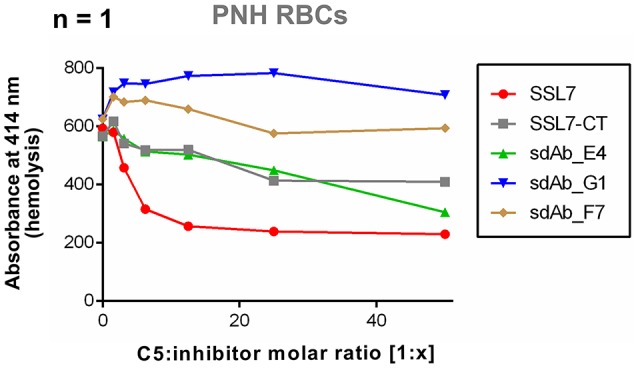
sdAb_E4 may prevent hemolysis of RBCs from PNH patients. Hemolysis of PNH erythrocytes by human serum in the presence of increasing concentrations of C5 inhibitor. The 100% hemolysis reference is taken from PNH RBCs incubated with human serum in the absence of inhibitor. Measurement were performed only once due to limited access to PNH erythrocytes.

Finally, to assess the properties of sdAb_E4 in a set up more relevant for therapeutic applications, we tested the effect of this sdAb on the lysis of human PNH erythrocytes by complement. Due to limited access to PNH erythrocytes, the experiment could unfortunately only be performed once. The results presented here are therefore only indicating a tendency. In the experiment conducted, exposition of fresh RBCs from a PNH patient to NHS induced RBC lysis, as measured by the release of hemoglobin (Figure 4). The presence of SSL7 prevented complement-mediated hemolysis and reached a plateau at a molar ratio of 1:12.5 (Figure 4). The residual lysis was in part due to spontaneous degradation of the RBCs during incubation. SSL7-CT and sdAb_E4 were also able to prevent complement-mediated RBC hemolysis (Figure 4), although they did not reach a plateau at molar ratios of up to 1:50 (Figure 4). Negative controls sdAb_G1 and sdAb_F7 did not prevent lysis mediated by complement activation on erythrocytes (Figure 4). These preliminary data suggest that sdAb_E4 may reduce complement-mediated lysis of RBCs from PNH patients. However, no firm conclusion can be drawn on the inhibitory role of sdAb_E4 from this single experiment and replicates will be necessary to conduct a more thorough analysis.

## Discussion

In this study, we used a pathogen mimicry strategy to achieve complement specific blockade, through the selection of an SSL7-like C5 inhibitor. The rationale for selecting such an inhibitor was based on conclusions derived from our structures of the C5:SSL7 and CVF:C5:SSL7 complexes (25, 40). We designed an antibody phage display protocol and succeeded in selecting a sdAb which behaved similarly to SSL7 in all our assays and which displayed specific properties toward the inhibition of TP. Hence, sdAb_E4 acts as a MAC-specific inhibitor, blocking the formation and deposition of the lytic complex on both endothelial cells and RBCs. Furthermore, we succeeded in reproducing the selectivity of the inhibition observed with SSL7-CT, i.e., our selected sdAb blocks CP- and AP-induced hemolysis while preserving almost completely the bactericidal activity of complement on *E. coli* cells, and to an even better extent than SSL7-CT. Although C5 cleavage in the fluid phase was nearly completely prevented by sdAb_E4 but also by both SSL7 and SSL7-CT (Figure 1D and Supplementary Figure 1), these molecules allowed partial C5 cleavage in the context of a pathogen membrane-bound C5 convertase (25). Indeed bactericidal capacity of human serum was still possible and our previous study on dead *S. aureus* showed that SSL7-CT maintained partial cleavage of C5 as compared to intact SSL7 (25). These results suggest that C5 cleavage in the fluid phase and on host cells surface, which can induce tissue damage, will be prevented by sdAb_E4, while still keeping the pathogen destruction capacity. Finally, preliminary tests on PNH RBCs suggest that our sdAb could prevent their lysis. Further experiments will however be necessary to conclude whether sdAb_E4 can efficiently block lysis of PNH erythrocytes. In any case, our study validates the feasibility of obtaining a sdAb with selective inhibitory properties toward complement TP using a structure-based strategy of pathogen mimicry.

Our sdAb_E4 candidate only displays medium affinity for human C5, with K_D_ value in the low micromolar range. This represents a 200-fold lower affinity for C5 as compared to the model molecule SSL7 (24). Such difference could notably explain the somewhat distinct performances of the sdAb and SSL7 in the hemolysis and bacteriolysis assays. As compared to the C5:eculizumab complex which displays affinity in the 100 pM range (29, 41), the strength of the C5:sdAb_E4 interaction is even lower (10^4^ to 10^5^ decrease in affinity). Such low affinity of sdAb_E4 for C5 as compared to already prescribed drugs obviously precludes its evaluation in animal disease models or even in clinical applications. Indeed, we already noticed in our assays that higher amounts of sdAb_E4 were generally needed to achieve similar inhibition than that observed with the SSL7 proteins. It is therefore probable that large amounts of sdAb_E4 would also be required *in vivo* to efficiently block complement TP. In this context, comparison of the performances of sdAb_E4 and eculizumab in our assays did not appear relevant to us since the results would be markedly biased by the huge affinity difference between the two molecules. A more affine sdAb is required to perform a detailed comparative analysis and to move on to *in vivo* studies. Nevertheless, the desired selectivity has been achieved with our current sdAb and, as such, it constitutes a promising scaffold for the selection, through affinity maturation, of an sdAb_E4-derived drug candidate that could be used in the treatment of complement-mediated hemolytic disorders.

As for SSL7-CT, the precise mode of action of sdAb_E4 remains unclear. For SSL7, two complementary modes of action have been proposed. The first one, IgA-dependent, relies on the simultaneous interaction of SSL7 with both IgA and C5, through SSL7 N- and C-terminal domains, respectively (25, 39, 40). This generates a large IgA-SSL7_2_-C5_2_ complex which impairs convertase binding to its substrate by steric hindrance (25), thereby annihilating all C5 functions. A second IgA-independent mechanism which applies for SSL7-CT must also take place, although its exact nature remains to be determined (25, 39). In particular, this second mechanism must account for differential SSL7 effects on bacteriolysis and hemolysis. It has been proposed that SSL7 on its own may modulate convertase binding to C5 (25). Structural studies of the CVF:C5 complex revealed that in the presence of SSL7, recognition of C5 by the convertase is somewhat weakened due to partial disturbance of the CVF(MG4):C5(MG5) interface by both the β-grasp and OB domains of the staphylococcal protein (40). A similar effect is predicted to occur with the endogenous C5 convertases and could be more pronounced on one or the other C5 convertases (CP/LP or AP convertase). This hypothesis could explain the results of our C5a release assay in which SSL7-CT impairs C5a production to almost the same extent as intact SSL7 (Figure 1D). In addition, SSL7 may also directly act on MAC inhibition, downstream of C5 cleavage. SSL7 can bind to C5b, which renders this hypothesis plausible (25). However, it is unclear whether the inhibitory effect would be linked to a direct competition for C5 binding between SSL7 and the C6-C9 proteins forming the MAC or whether another mechanism is involved. Recent structures of the C5b-C6 complex did not reveal any overlap between the SSL7- and C6-binding sites on C5b (42, 43), but the binding of other MAC components may be hindered by SSL7, either directly or through allosteric movements. The recent cryo-electron microscopy map of the MAC refined at a resolution of 8.5 Å seems to position the two CCP domains of complement C7 in the cavity delineated by C5 MG1-MG2 and MG5-MG6 domains (44). SSL7 could therefore potentially interfere with the recruitment of C7 on the C5b-C6 complex. Finally, regardless of the inhibitory mechanism employed by SSL7, another possible explanation for its differential inhibition of hemolysis and bacteriolysis is that MAC assembly on RBCs and bacterial cells may occur through distinct mechanisms that would be impaired differentially by the presence of SSL7 (25).

Extrapolation of all these models to our anti-C5 sdAb is clearly possible since it behaves similarly to SSL7-CT in all the assays we tested. Thus, as proposed for SSL7, sdAb_E4 may affect differently C5 convertases binding to their C5 substrate and/or the formation of MAC on RBCs vs. Gram negative bacteria. The fact that sdAb_E4 proves to be more potent than SSL7-CT in maintaining efficient bacteriolysis may be linked to its reduced affinity for C5 as compared to SSL7 (24), which is also consistent with the fact that higher concentrations of sdAb_E4 are needed to reach the same degree of hemolysis inhibition as compared to the SSL7 fragments (Figures 3A,B). sdAb_E4 may therefore act as a MAC modulator rather than an inhibitor, which appears to be advantageous in this context. In the absence of a crystal structure for the C5:sdAb_E4 complex, we cannot exclude that the binding epitope of sdAb_E4 on C5 differs from that of SSL7. Our attempts to crystallize the complex have so far remained unsuccessful, possibly due to the low affinity of the sdAb for its target. Another explanation could be the too fast kinetics binding parameters of the C5:sdAb_E4 interaction, leading to a highly dynamic complex which tends to dissociate rapidly, thus failing to crystallize. A more affine sdAb is probably necessary to conduct such structural studies and gain detailed insights on the inhibitory mechanism of sdAb_E4. In this perspective, an immune sdAb library may have proven more advantageous than the synthetic GARVAN library we used. Indeed, affinity maturation inherent to *in vivo* immunization may have allowed us to obtain sdAbs of much higher affinities than the ones obtained from a synthetic library. Obviously, we cannot rule out that sdAb_E4 binds to other components in human serum, although its mode of selection (phage display against C5 and elution with SSL7) suggests that the antibody should be highly specific for the C5 molecule. More detailed studies are clearly needed to fully characterize the inhibitory mode of our selected sdAb. On the other hand, these mechanistic studies will be more informative once a more affine sdAb candidate has been obtained, the current study only demonstrating the validity of our drug-design approach.

The effectiveness of C5 inhibition in PNH and aHUS opens up a gateway for testing this strategy in a large panoply of other diseases, as diverse as graft rejection, sickle cell disease or even cancer, where complement contributes to pathogenesis (22, 34, 45). Our study provides a proof of concept for the use of SSL7 mimicry as a principle for specific inhibition of complement, and opens up promising perspectives for the development of therapeutic molecules targeting complement-mediated pathologies.

## Author contributions

PK and GA conceived the original research project. LY, MB, LR, ST, PK, and GA designed the experiments. LY, NM, AH, NF, JØ, and MB performed the experiments. LY, LR, ST, and GA analyzed the data. LY and GA wrote the manuscript with input and approval from all co-authors.

### Conflict of interest statement

GA declares a collaboration with Alexion pharmaceuticals. The remaining authors declare that the research was conducted in the absence of any commercial or financial relationships that could be construed as a potential conflict of interest.

## Abbreviations

aHUS: atypical hemolytic uremic syndrome
AP: alternative pathway of the complement system
BLI: biolayer interferometry
CP: classical pathway of the complement system
CVF: cobra venom factor
IPTG: isopropyl β-D-1-thiogalactopyranoside
MAC: membrane attack complex
MST: microscale thermophoresis
NHS: normal human serum
sdAb: single-domain antibody
PNH: paroxysmal nocturnal hemoglobinuria
SEC: size exclusion chromatography
SSL7: staphylococcal superantigen-like protein 7
TEV: Tobacco Etch virus
TMB: 3,3′,5,5′-Tetramethylbenzidine
TP: terminal pathway of the complement system.
