# Supplementary material for: A Single-Domain Antibody Targeting Complement Component C5 Acts as a Selective Inhibitor of the Terminal Pathway of the Complement System and Thus Functionally Mimicks the C-Terminal Domain of the Staphylococcus aureus SSL7 Protein
Source: Front Immunol. 2018 Nov 29;9:2822. doi: 10.3389/fimmu.2018.02822 (PMC6281825; doi:10.3389/fimmu.2018.02822)
Supplement: Supplementary file 1 [file Data_Sheet_1.pdf]

Supplementary Material

A Single-Domain Antibody Targeting Complement Component C5 Acts as a Selective Inhibitor of the Terminal Pathway of the Complement System and Thus Functionally Mimicks the C-Terminal Domain of the *Staphylococcus aureus* SSL7 Protein

Laure Yatime[1,6*], Nicolas S. Merle[2], Annette G. Hansen[3], Niels Anton Friis[1,7], Jakob A. Østergaard[4], Mette Bjerre[4], Lubka T. Roumenina[2], Steffen Thiel[3], Peter Kristensen[5], Gregers R. Andersen[1]

[1]Department of Molecular Biology and Genetics, Aarhus University, Aarhus, Denmark

[2]Centre de Recherche des Cordeliers, INSERM, UMR_S 1138, Paris, France

[3]Department of Biomedicine, Aarhus University, Aarhus, Denmark

[4]The Medical Research Laboratory, Department of Clinical Medicine, Aarhus University and Department of Endocrinology and Internal Medicine, Aarhus University Hospital, Aarhus, Denmark

[5]Department of Chemistry and Bioscience, Aalborg University, Aalborg, Denmark

[6]Present address:

DIMNP – UMR5235, CNRS, INSERM, University of Montpellier, Montpellier, France

[7]Present address:

Biotest Facility ApS, Trige, Denmark

*** Correspondence:**
Dr. Laure Yatime
laure.yatime@inserm.fr

CONTENTS

**Supplementary Figure 1.**	C5a release assay in human serum
**Supplementary Figure 2.**	Evaluation of the affinity between human C5 and sdAb_E4 using biolayer interferometry (BLI)
**Supplementary Figure 3.**	Hemolysis assay on sheep RBCs

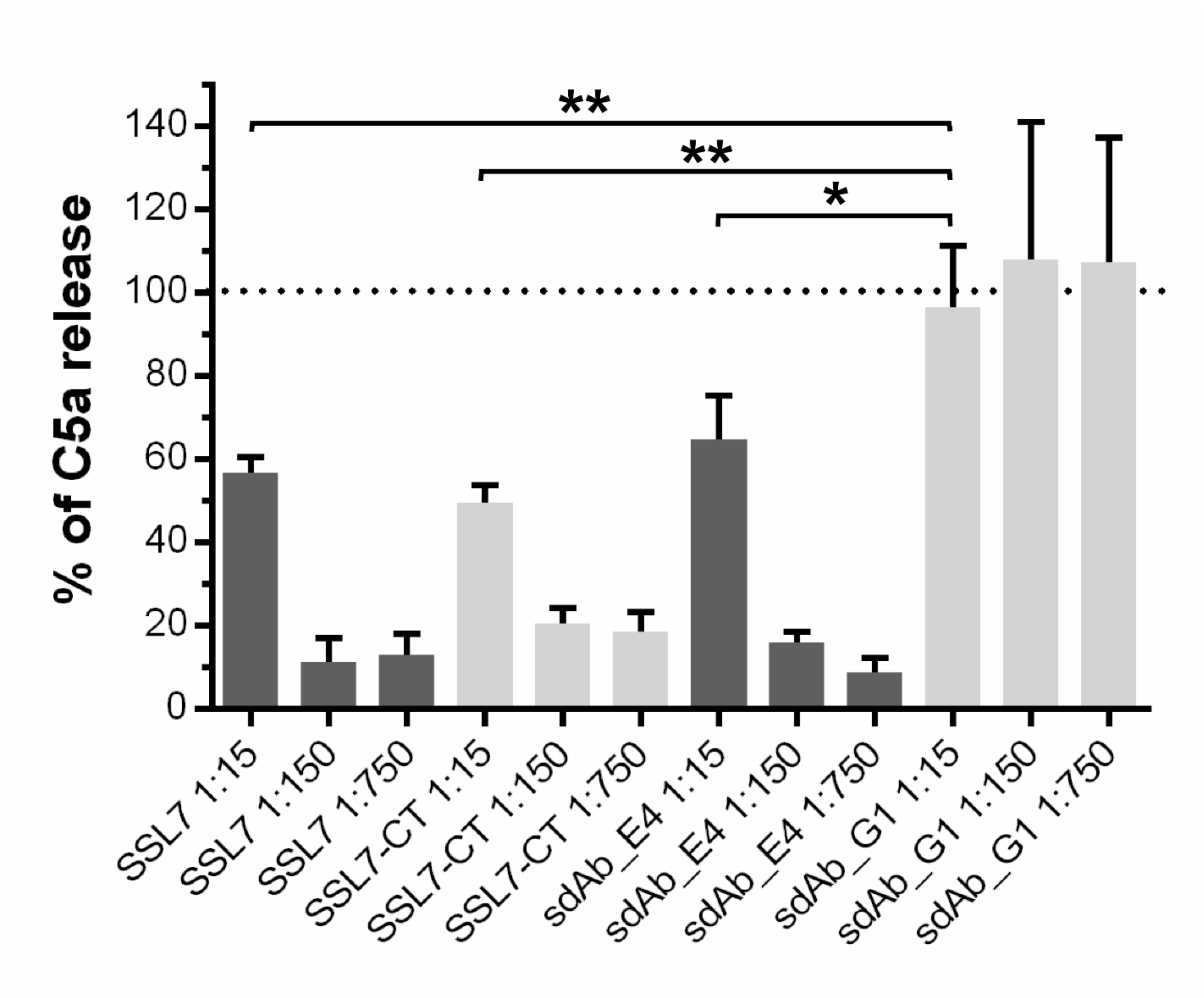

Supplementary Figure 1. C5a release assay. Measurement of the amount of C5a released in human serum upon activation at 37ºC in the presence of different concentrations of C5 inhibitors (same as Figure 1D in the main text but with C5:inhibitor molar ratios extending to 1:750). The residual amount of C5a released in the absence of activation at 37ºC was subtracted from all sample values. The 100% reference is taken as the value obtained with NHS incubated at 37ºC in the absence of any inhibitor. P-values: * $p < 0.05$; ** $p < 0.01$.

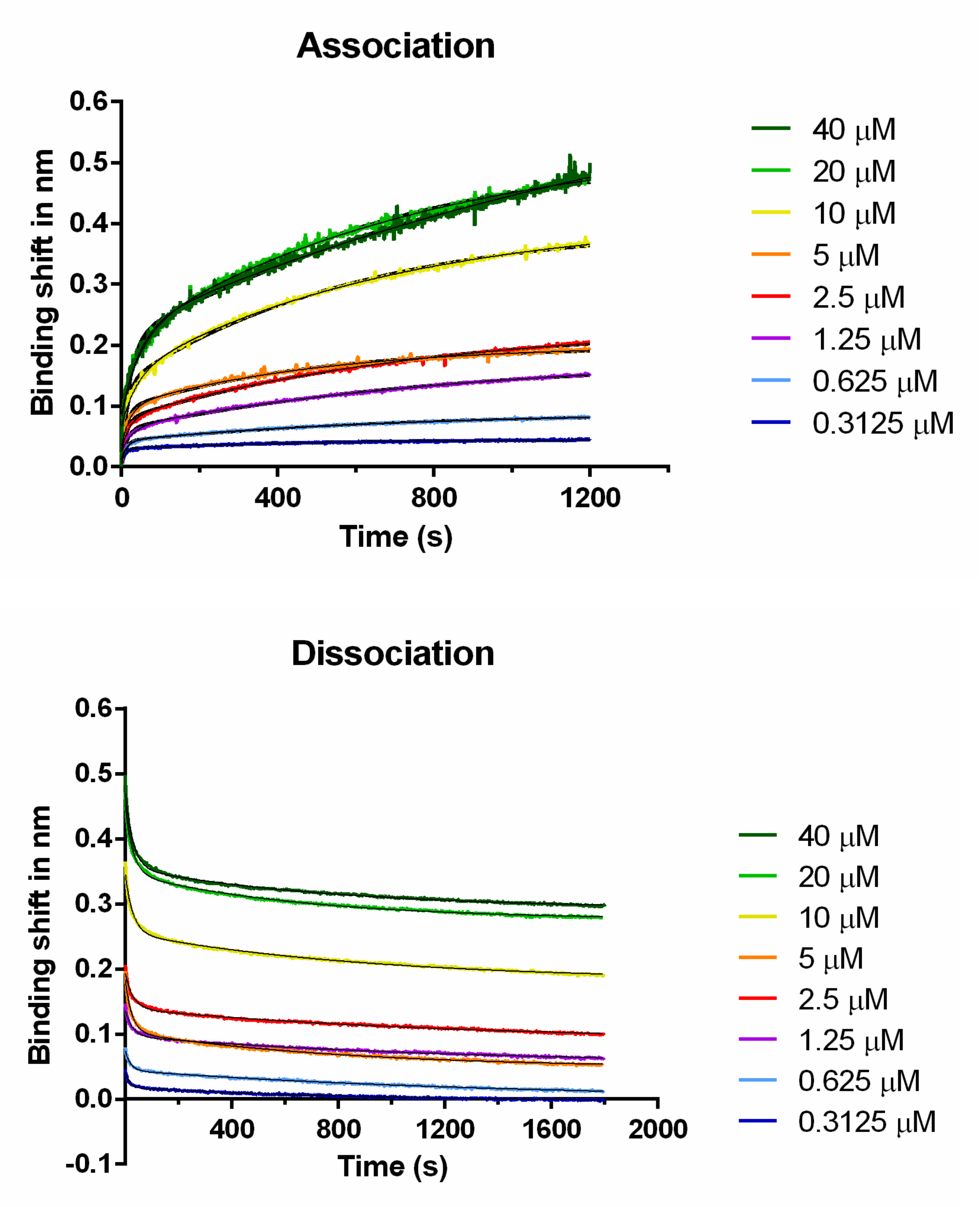

Supplementary Figure 2. Evaluation of the affinity between human C5 and sdAb_E4 using biolayer interferometry (BLI). BLI sensorgrams obtained for the association phase and the dissociation phase for complex formation between immobilized sdAb_E4 and human C5 as the analyte in solution. Curve fitting was performed with GraphPad Prism (fitting curves shown in black on the sensorgrams) and yielded K_D values ranging between 1 and 12 μM for the various datasets.

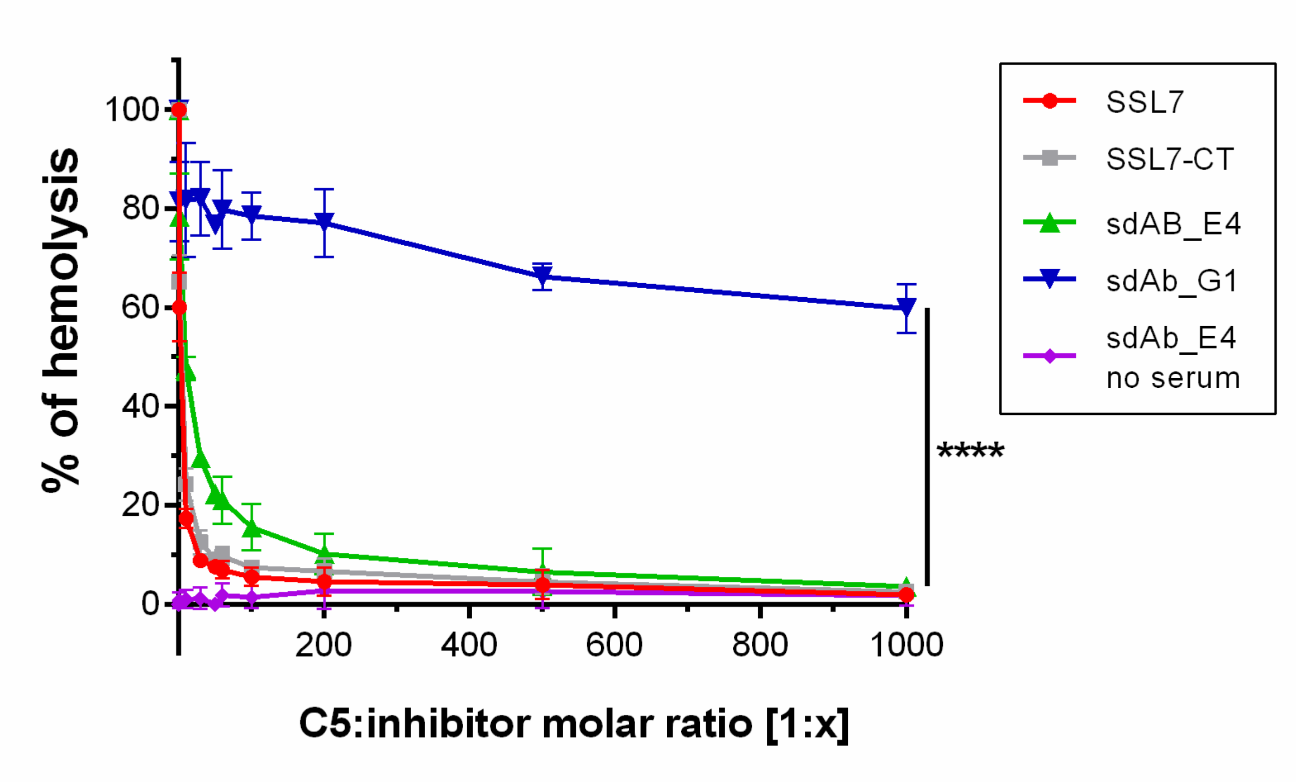

Supplementary Figure 3. Hemolysis assay on sheep RBCs. Same as Figure 3A from main text but with data extending to a C5:inhibitor molar ratio of 1:1000. P-values: **** $p < 0.0001$.
